# Visualization of metabolite distribution based on matrix-assisted laser desorption/ionization–mass spectrometry imaging of tea seedlings (*Camellia sinensis*)

**DOI:** 10.1093/hr/uhae218

**Published:** 2024-08-03

**Authors:** Maoyin Fu, Liying Tian, Dongqiao Zheng, Yang Gao, Chenyi Sun, Shihua Zhang, ZhaoLiang Zhang, Xiaochun Wan, Qi Chen

**Affiliations:** State Key Laboratory of Tea Plant Biology and Utilization, School of Tea and Food Science & Technology, Anhui Agricultural University, Hefei 230036, China; State Key Laboratory of Tea Plant Biology and Utilization, School of Tea and Food Science & Technology, Anhui Agricultural University, Hefei 230036, China; State Key Laboratory of Tea Plant Biology and Utilization, School of Tea and Food Science & Technology, Anhui Agricultural University, Hefei 230036, China; State Key Laboratory of Tea Plant Biology and Utilization, School of Tea and Food Science & Technology, Anhui Agricultural University, Hefei 230036, China; State Key Laboratory of Tea Plant Biology and Utilization, School of Tea and Food Science & Technology, Anhui Agricultural University, Hefei 230036, China; College of Computer Science, South-Central Minzu University, Wuhan 430074, China; State Key Laboratory of Tea Plant Biology and Utilization, School of Tea and Food Science & Technology, Anhui Agricultural University, Hefei 230036, China; State Key Laboratory of Tea Plant Biology and Utilization, School of Tea and Food Science & Technology, Anhui Agricultural University, Hefei 230036, China; State Key Laboratory of Tea Plant Biology and Utilization, School of Tea and Food Science & Technology, Anhui Agricultural University, Hefei 230036, China; Key Laboratory of Food Nutrition and Safety, Anhui Engineering Laboratory for Agro-products Processing, School of Tea and Food Science & Technology, Anhui Agricultural University, Hefei 230036, China

## Abstract

Tea seedlings (*Camellia sinensis*) have a well-developed root system with a strong taproot and lateral roots. Compared with ordinary cuttings, tea has stronger vitality and environmental adaptability, thus facilitating the promotion of good varieties. However, there is less of detailed research on the rooting and germination process of tea seeds. In this study, matrix-assisted laser desorption ionization time-of-flight–mass spectrometry was used to conduct non-targeted spatial mass spectrometry imaging of the main organs during growth of tea seedlings. A total of 1234 compounds were identified, which could be divided into 24 classes. Among them, theanine, as the most prominent nitrogen compound, was synthesized rapidly at the early stage of embryo germination, accounting for >90% of the total free amino acids in the radicle, and it was then transferred to each meristem region through the mesocolumnar sheath, indicating that theanine-based nitrogen flow plays a decisive role in organ formation during the development of tea seedlings. Nutrients stored in the cotyledon were rapidly hydrolyzed to dextrin and 3-phosphoglyceraldehyde at the early stages of germination, and subsequently converted to other forms that provided carbon and energy for development, such as raffinose and d-galactose (glucose), which were mainly distributed in the growing zones of the root apex and the apical meristems of the stem. This study provides a new perspective on the synthesis and metabolism of substances during the development of tea seedlings and contributes to a better understanding of the biological characteristics of tea varieties.

## Introduction

The tea tree [*Camellia sinensis* (L.) O. Kuntze] originated in China and is a perennial woody plant belonging to the section *Thea* (L.) Dyer. Tea tree is an important leafy cash crop, and tea is one of the most popular non-alcoholic beverages in China [1]. Tea leaves are made from the leaves of the tea tree after harvesting through various processes, and they are rich in secondary metabolites, including amino acids, catechins, caffeine, and volatile aroma compounds [2]. These substances not only contribute to the unique flavor of tea, but they are also very beneficial to human health, with properties such as anti-disease and memory improvement effects [3–5]. Therefore, the development and utilization of the functional components of the tea tree has become a research hot spot in recent years.

Nitrogen is an important factor regulating plant growth and development and the synthesis of secondary metabolites, and amino acids are an important expression of nitrogen in plants, as well as the main form of nitrogen transport and redistribution over long distances [6]. Amino acids are not only involved in protein synthesis in plants, but they are also precursors of endogenous plant hormones, chlorophyll, polyamines, and many important secondary metabolites [7, 8]. In addition, amino acids play many roles in plants, including acting as signaling molecules and regulating root and shoot development as well as flowering time and abiotic stress responses [9]. In the tea tree, free amino acids also contribute to the formation of aroma compounds and other secondary metabolites necessary for tea tree growth and stress adaptation [10]. The main forms of amino acids in tea tree include theanine (Thea), arginine (Arg), glutamate (Glu), and glutamine (Gln) [11]. It has been shown that Thea is a unique non-protein amino acid in tea tree that accounts for 60–70% of the total free amino acids in the new tips of tea tree [12], and it is an important form of free nitrogen in tea tree. However, the distribution and function of Thea in the growth and development of tea tree have yet to be studied in depth.

In plants, sugars not only provides energy for plant growth and development [13] through starch metabolism, the glycolytic pathway, and the tricarboxylic acid (TCA) cycle, regulates the carbon and nitrogen balance, and forms the underlying carbon skeleton, but also serves as an important signal molecule, regulating organ development and formation in conjunction with phyrohormones [14]. They also participate in the formation of plant secondary metabolites as the main carbon skeleton. At the same time, the carbon metabolic flow is also influenced by plant nitrogen nutrition, which can provide the necessary carbon skeleton for processes such as nitrogen assimilation and amino acid synthesis by increasing the synthesis of organic acids in the plant [15, 16]. The effects of carbon metabolic flow also include reducing the starch content, affecting the hormone level, reducing the root/crown ratio, and delaying flowering [17–19].

Currently, research on these metabolites in plants is mainly conducted using chromatography–mass spectrometry, which combines the efficient separation ability of chromatography and the powerful analysis function of mass spectrometry, and it is an important technical tool for the study of metabolites [20–22]. However, the process of grinding tissue homogenates required for analysis using this technique results in the loss of information on the spatial distribution of metabolites in tissues, which is not conducive to the investigation of the mechanism of metabolite formation at the tissue and cellular levels. The mass spectrometry image (MSI) technique is a new molecular imaging technique combining mass spectrometry and 2D spatial imaging that can be used for both quantitative detection and qualitative analysis. In the field of MSI, matrix-assisted laser desorption ionization time-of-flight mass spectrometry imaging (MALDI-TOF-MS), secondary ion mass spectrometry (SIMS-MSI), desorption electrospray ionization mass spectrometry imaging (DESI-MSI), and laser sputtering electrospray ionization mass spectrometry imaging (LAESI-MSI) are the most widely used MSI techniques for the visualization of plant secondary metabolites [23, 24]. MALDI-TOF-MS technology is widely used in precision medicine, translational medicine, clinical research, and basic life science research because of its high sensitivity, high resolution, fast imaging speed, complete spatial information retention, lack of chemical or radioactive labeling, and lack of complicated sample pre-treatment [25]. With the maturation of MSI technology in recent years, an increasing number of spatial metabolomics studies on plants have been conducted. For example, Wang *et al*. [26] used MALDI-TOF-MS to map the spatial profiles of metabolites in strawberries at four different stages of ripening and characterized the differences in the distribution of secondary metabolites anthocyanins, TCA cycle products citric acid, and soluble sugars. Zhao *et al*. [27] discovered the spatial distribution of endogenous molecules, such as choline, betaine, citric acid, hexose, and sucrose, during the development of *Lycium barbarum* using the MALDI-MSI technique. Using a combination of MALDI-MSI and liquid chromatography–mass spectrometry, Montini *et al*. [28] revealed that dhurrin and its recycling products accumulated mainly in the germinating embryo, shield, and seed coat regions during sorghum development [28]. Xia *et al*. [29] used multi-omics and DESI-MSI to reveal the spatial distribution and molecular mechanisms of terpenoid biosynthesis in *Salvia miltiorrhiza* and *Salvia grandifolia* [29]. In tea tree, Liao *et al*. [30] described for the first time the spatial distribution of l-Thea and l-Val in the extra-root cross-section of tea leaves using DESI-MSI and found that compounds such as catechins (ECG, CG, EGCG, GCG, GCG) were almost uniformly distributed on both sides of the tea leaves, whereas EC, C, EGC, GC, and gallocatechin A were distributed near the leaf veins. However, there are no relevant reports on the spatial and temporal distribution of metabolites in different organs during the development of tea seedlings. In this study, MALDI-MSI combined with non-targeted spatial metabolomics analysis was used to map the spatial and temporal distribution of metabolites in different organs of tea seedlings, so as to analyze the key substances affecting the growth of tea seedlings and explore their synthesis mechanism. The aim of this study was to analyze the biological characteristics of tea seedlings during their germination through spatial metabolomics, and to provide a new perspective for the study of special nitrogen metabolism mechanism of the tea plant centred on Thea.

## Results

### Classification of substances detected in samples from various organs during different developmental stages of tea seedlings

**Figure 1:**
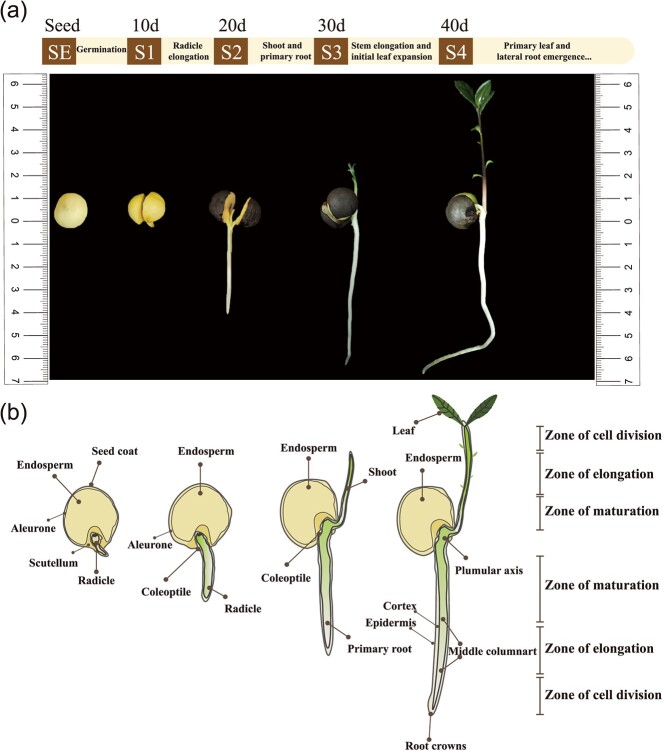
Tea seedling development process and sampling time point. **a** Samples of tea seedlings at different developmental periods: SE, seed; S1, seed germination and exposure of radicle growth point (10 days); S2, first growth of radicle (20 days); S3, growth of shoots (30 days); S4, growth of true leaves (40 days). **b** Schematic of longitudinal sections of the four developmental stages selected for MSI analysis, showing detailed morphological features and main compartment**s**.

In this experiment, a non-targeted spatial metabolome approach was used for comprehensive metabolite detection in different organs (Fig. 1b) of tea seedlings during different developmental periods (Fig. 1a). Supplementary Data Fig. S1 describes in detail the process of sample preparation and spatial mass spectrometry (MS) detection. Samples from different tissues at different developmental stages of tea seedlings (Supplementary Data Fig. S2) were pre-cooled and embeded in OCT(optimal cutting temperature compound) for freezing, then sliced, and appropriate slices were selected to spray substrates, followed by MALDI-MS detection. The results was used to express the relative quantitative values of metabolites in different regions in the embryo, radicle, and germ organs, after root mean square normalization of the peak intensity values of the target peaks, and the peaks obtained from the labeled peaks. The target peaks with higher intensities (MS primary information) were fragmented *in situ* in tissues to obtain secondary profiles (MS/MS secondary fragmentation peak information), and the secondary profiles collected from tissues were compared by searching the self-built database and integrating the public library (HMDB) for substance identification. Finally, 335 substances were identified based on MS/MS secondary fragment peak information (Supplementary Data Table S1). The remaining target peaks with lower intensities for which secondary MS information could not be collected were searched according to their primary molecular weights (MS primary information) within a 10-ppm error range by searching the self-built database and integrating the substance search of the public library (HMDB). The substances with molecular weights closest to those detected using the instrument were screened, and a total of 899 substances were identified (Supplementary Data Table S2). Supplementary Data Fig. S3 shows the mean mass spectrum of MALDI in the range of *m/z* 50–1300 obtained from different regions during the development of tea seedlings in the positive ion mode, and Supplementary Data Fig. S3b shows some representative secondary metabolites of the tea plant.

**Figure 2:**
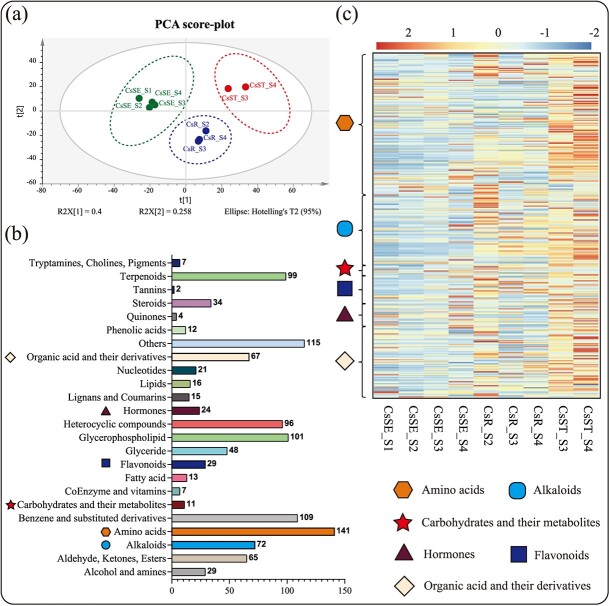
Metabolomics analysis of all samples. **a** PCA analysis of the samples. **b** Classification of all metabolites detected in the samples. **c** Changes in the relative amounts of selected metabolites in all samples. CsSE, cotyledon; CsR, root tip; CsST, shoot.

The types of metabolites detected could be classified into 24 categories, including amino acids, terpenoids, alkaloids, flavonoids, benzene and its derivatives, carbohydrates, phenolic acids, lipids, and hormones (Fig. 2b). The leaves at S4 stage were not examined for the spatial metabolome because it was difficult to obtain ideal slides by sectioning the leaf parts. Principal component analysis (PCA) was performed for all of the compounds in the embryo (four stages, S1–S4), root tip (three stages, S2, S3, and S4), and stem tip (two stages, S3 and S4) parts during the development of tea seedlings. As seen in Fig. 2a, all of the samples could be clearly distinguished by organ, indicating a clear tissue-specific distribution of metabolites early in the development of tea seedlings. It has been shown that the biosynthesis of carbohydrates and amino acids, hormone signaling, glycolysis, the TCA cycle, and some alkaloids and flavonoids have important roles in seed germination and seedling growth [31, 32]. The relative contents of amino acids, flavonoids, alkaloids, carbohydrates, hormones and hormone-related compounds, and organic acids and their derivatives in different tissues were selected for display in the heat map shown in Fig. 2c. It was obvious that the metabolite changes gradually became active with the growth of tea seedlings, and the relative contents of these metabolites in tea seed cotyledons were significantly lower than those in root and stem tips. In particular, the young root tips (CsR_S2) and young stem buds (CsST_S3, CsST_S4) had more abundant metabolites, probably because the young root and stem tips had more vigorous organ differentiation, which promoted the synthesis of metabolites in large quantities. The high contents of amino acids and organic acid metabolites during seed germination (CsSE_S1) may be related to protein degradation and starch hydrolysis in the endosperm of tea seeds during germination (Fig. 2c), and provide nitrogen nutrients and energy for the subsequent growth and development of tea seedlings through the processes of glycolysis and the TCA cycle. Although a few types of hormones were detected and their relative contents were low, they have an important influence on the growth and development of tea seedlings.

### Differential accumulation of free amino acid content in different tissues of tea seedlings during different developmental periods

A previous study showed that although the highest percentage of Thea content was found in the new tips, it was mainly synthesized in the roots [1]. To investigate the distribution pattern of nitrogen nutrients, during the development of tea seedlings, the present study measured the concentration of free amino acids and the ethylamine contents in different parts of tea seedlings during the SE, and S1–S4 periods (Table 1). The results showed that the free amino acid content varied drastically during the overall development of tea seedlings. The content of Thea was significantly higher than that of other amino acids, reaching a maximum of 88.07% of total free amino acids in roots, 90.08% in stems, and 81.68% in leaves during the S4 period, indicating that the development of tea seedlings during the S1–S4 periods was mainly dependent on Thea for nitrogen nutrition. More interestingly, the Thea content was low in the seed budding stage (1.419 mg/g, CsSE), but increased extremely significantly with the growth of the radicle (90.989 mg/g, CsR_S2), and reached a maximum in the roots (157.687 mg/g, CsR_S3) when the radicle grew out, at which time the Thea content in the epicotyl also reached its highest value of 88.316 mg/g (CsST_S3), indicating that Thea synthesis was strongly induced by the developmental signal of tea seeds. Subsequently, Thea content in roots and stems gradually decreased with seedling growth and tissue differentiation, indicating that Thea was involved in nitrogen utilization in aboveground organ formation. In addition, the contents of Glu and Gln, the synthetic precursors of Thea, showed the same trend as that of Thea in the roots, both of which increased and then decreased with the growth and development of tea seedlings. The contents of arginine, aspartic acid (Asp), alanine (Ala), and serine, which are more abundant in all organs of tea seedlings at S1–S4 stages, show similar changes in developmental stages.

**Table 1:** Free amino acid content of different tissues of tea seedling during SE–S4 (mg/g, DW).

Component	CsSE	CsR_S1	CsR_S2	CsR_S3	CsR_S4	CsST_S3	CsST_S4	CsL_S4
Thea	1.419 ± 0.466^d^	6.984 ± 2.637^d^	90.989 ± 55.375^b^	157.687 ± 20.153^a^	104.319 ± 25.781^b^	88.316 ± 27.308^b^	69.812 ± 13.349^bc^	32.008 ± 2.068^cd^
Ala	0.165 ± 0.061^e^	0.742 ± 0.299^a^	0.470 ± 0.209^abcd^	0.554 ± 0.114^abc^	0.573 ± 0.043^ab^	0.297 ± 0.105^bcde^	0.281 ± 0.022^cde^	0.203 ± 0.049^de^
Val	0.102 ± 0.011^bc^	0.184 ± 0.076^a^	0.141 ± 0.026^ab^	0.151 ± 0.020^ab^	0.159 ± 0.027^ab^	0.098 ± 0.018^bc^	0.103 ± 0.023^bc^	0.067 ± 0.006^c^
Leu	0.039 ± 0.019^bc^	0.152 ± 0.060^a^	0.074 ± 0.059^b^	0.056 ± 0.008^bc^	0.061 ± 0.013^bc^	0.028 ± 0.012^bc^	0.033 ± 0.019^bc^	0.008 ± 0.008^c^
Ser	0.164 ± 0.066^e^	0.358 ± 0.106^e^	1.471 ± 0.957^bcd^	3.531 ± 0.489^a^	2.454 ± 0.650^b^	1.779 ± 0.620^bc^	1.408 ± 0.308^cd^	0.640 ± 0.162^de^
Gly	0.014 ± 0.001^c^	0.054 ± 0.027^b^	0.068 ± 0.021^ab^	0.096 ± 0.033^a^	0.063 ± 0.009^ab^	0.039 ± 0.014^bc^	0.038 ± 0.006^bc^	0.044 ± 0.010^bc^
Cys	0.042 ± 0.002^b^	0.093 ± 0.094^b^	0.551 ± 0.334^a^	0.785 ± 0.061^a^	0.473 ± 0.103^a^	0.711 ± 0.212^a^	0.579 ± 0.100^a^	0.776 ± 0.204^a^
Asp	1.274 ± 0.240^ab^	1.502 ± 0.163^ab^	1.503 ± 1.020^ab^	1.772 ± 0.321^a^	1.736 ± 0.287^a^	0.569 ± 0.510^b^	0.665 ± 0.316^b^	0.822 ± 0.279^ab^
Met	ND	0.017 ± 0.018^a^	0.015 ± 0.017^a^	ND	ND	0.016 ± 0.015^a^	0.002 ± 0.004^a^	0.015 ± 0.013^a^
Thr	0.087 ± 0.022^d^	0.244 ± 0.082^bcd^	0.286 ± 0.170^bc^	0.516 ± 0.082^a^	0.435 ± 0.132^ab^	0.241 ± 0.081^bcd^	0.254 ± 0.102^bcd^	0.098 ± 0.022^cd^
Lys	0.133 ± 0.026^abc^	0.150 ± 0.139^ab^	0.083 ± 0.049^bc^	0.151 ± 0.020^ab^	0.221 ± 0.039^a^	0.063 ± 0.016^bc^	0.087 ± 0.009^bc^	0.027 ± 0.007^c^
Ile	0.008 ± 0.008^bc^	0.069 ± 0.039^a^	0.034 ± 0.014^bc^	0.026 ± 0.002^bc^	0.038 ± 0.013^ab^	0.017 ± 0.006^bc^	0.024 ± 0.012^bc^	0.003 ± 0.005^c^
Glu	1.827 ± 0.204^b^	2.098 ± 1.345^b^	3.157 ± 2.002^ab^	4.469 ± 0.205^a^	3.411 ± 0.613^ab^	2.061 ± 0.603^b^	2.054 ± 0.239^b^	2.814 ± 0.227^ab^
Gln	0.451 ± 0.246^b^	1.624 ± 0.705^ab^	4.145 ± 2.506^a^	2.551 ± 1.975^ab^	2.861 ± 2.861^ab^	1.251 ± 0.827^ab^	1.986 ± 1.658^ab^	0.694 ± 0.661^b^
Pro	0.007 ± 0.008^b^	0.104 ± 0.044^a^	0.042 ± 0.037^b^	0.007 ± 0.006^b^	0.007 ± 0.012^b^	0.007 ± 0.007^b^	0.003 ± 0.005^b^	0.007 ± 0.010^b^
Hypro	ND	0.008 ± 0.005^a^	ND	ND	ND	ND	ND	ND
Orn	ND	ND	0.048 ± 0.018^a^	0.137 ± 0.020^a^	0.125 ± 0.045^a^	0.093 ± 0.044^a^	0.073 ± 0.017^a^	0.082 ± 0.077^a^
Cit	0.014 ± 0.012^c^	0.046 ± 0.018^bc^	0.166 ± 0.101^ab^	0.164 ± 0.046^ab^	0.284 ± 0.123^a^	0.099 ± 0.047^bc^	0.091 ± 0.012^bc^	0.093 ± 0.009^bc^
Arg	6.007 ± 1.296^b^	7.084 ± 2.546^b^	1.511 ± 1.260^de^	4.927 ± 0.204^bc^	11.819 ± 1.662^a^	1.772 ± 0.280^de^	3.344 ± 0.895^cd^	0.496 ± 0.225^e^
Phe	0.264 ± 0.060^a^	0.302 ± 0.727^a^	0.077 ± 0.026^b^	0.049 ± 0.005^b^	0.032 ± 0.009^b^	0.042 ± 0.013^b^	0.044 ± 0.008^b^	0.102 ± 0.058^b^
Tyr	ND	0.060 ± 0.032^ab^	ND	0.011 ± 0.019^c^	0.080 ± 0.023^a^	0.008 ± 0.013^c^	0.031 ± 0.027^bc^	0.024 ± 0.021^bc^
His	0.023 ± 0.021^c^	0.193 ± 0.079^bc^	0.631 ± 0.489^a^	0.639 ± 0.218^a^	0.579 ± 0.132^ab^	0.112 ± 0.032^c^	0.104 ± 0.002^c^	0.027 ± 0.027^c^
GABA	0.029 ± 0.004^c^	1.059 ± 0.695^a^	0.564 ± 0.207^abc^	0.766 ± 0.107^ab^	0.758 ± 0.159^ab^	0.423 ± 0.098^bc^	0.538 ± 0.045^abc^	0.137 ± 0.035^c^
Total amino acids	12.067	23.128	106.028	179.046	130.491	98.041	81.555	39.188
Theanine proportion (%)	11.76	30.20	85.82	88.07	79.94	90.08	85.60	81.68
Ethylamine	ND	0.121 ± 0.038^c^	0.501 ± 0.077^ab^	0.687 ± 0.097^a^	0.411 ± 0.201^ab^	0.489 ± 0.286^ab^	0.461 ± 0.040^ab^	0.362 ± 0.057^bc^

CsSE, seed; CsR_S1, root during S1 stage; CsR_S2, root during S2 stage; CsR_S3, root during S3 stage; CsR_S4, root during S4 stage; CsST_S3, stem during S3 stage; CsST_S4, stem during S4 stage; CsL_S4, true leaf during S4 stage. ND, not detected. Values are means±SD, and lower-case letters denote significant differences in Duncan’s multiple extreme difference test (*P* < 0.05).

This study also determined the content of ethylamine, another Thea synthesis precursor, using gas chromatography–mass spectrometry (GC–MS). The trend of ethylamine content was more consistent with that of Thea, with a correlation of 0.71 between the two at the root (*P* = 0.0095); ethylamine increased significantly in the roots during the S1–S2 periods and reached the highest content in the roots (0.687 mg/g) and stem buds (0.489 mg/g) during the S3 period. However, ethylamine was not detected in tea tree seeds (CsSE). In addition, in order to explore the reason for such high accumulation of theanine in roots, we calculated the expression levels of genes related to theanine and ethylamine synthesis. Supplementary Data Fig. S4 shows that the gene expression of theanine synthetase (*CsTSI*) maintained a high level at all stages of root development, and was not correlated with the changes of theanine and ethylamine (*R* = −0.18, *P* = 0.57; *R* = −0.012, *P* = 0.97). On the contrary, the expression level of alanine decarboxylase (*CsAlaDC*) in root was consistent with the contents of theanine and ethylamine, showing a significant positive correlation (*R* = 0.9, *P* = 6.4e^−05^; *R* = 0.84, *P* = 0.00059). The results showed that CsAlaDC, a key enzyme for the synthesis of ethylamine, was obviously induced in tea seedling development, while CsTSI was more affected by the change of the content of ethylamine. In addition, the expression of *CsGS* family members also showed differentiation, and the change trend of *CsGS2.1.1*and *CsGS2.1.2* was highly similar to that of theanine and ethylamine, which were induced by the development process, while the change of *CsGS2.1.3* expression was not obvious.

### MALDI-TOF MS study of theanine-based nitrogen metabolism compounds

**Figure 3:**
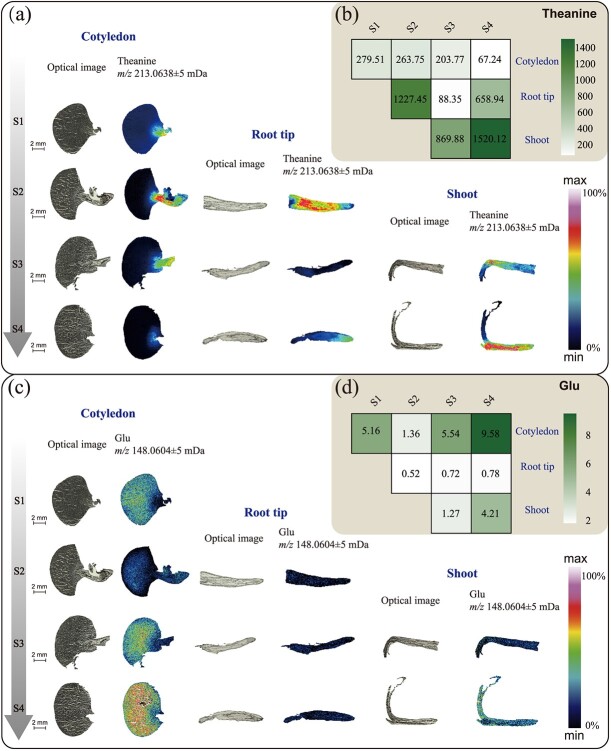
Spatial MSI distribution and relative contents of theanine and glutamate. **a**, **c** Optical images (left) and ion distribution images (right) of theanine and glutamate in cotyledons, root tips, and stems (leaf buds) at four developmental periods. The color scale from dark blue to purple represents increasing contents of the target substances sequentially in the region. **b**, **d** Heat maps of the relative contents of theanine and glutamic acid in different samples. The color scale indicates a sequential increase in the relative contents from white to green.

In the present study, Thea (*m/z* 213.0638, [M + K]^+^) was detected in different tissues of tea seedlings at different developmental stages, except in the endosperm, where it is almost undetectable. Large amounts of Thea were detected near the embryonic axis, root tip meristem, and shoot stems of tea seedlings, as shown in the heat map in Fig. 3a (in which colors from black to blue, cyan, and red represent increasing content of target substances in the region). During tea seedling development, the MS signal of Thea appeared for the first time in the embryo and hypocotyl at the seed germination stage (S1). During S2, the radicle of tea seedlings began to develop and elongate (mainly the meristem), and large amounts of Thea signals were detected in the roots, especially in the middle columnar sheath. Thea signals in the cotyledon and roots became significantly weaker in the S3 stage. As can be seen in Fig. 3b, with the growth of the epiblast, the Thea content in the roots decreased to the lowest level, at which time the signal intensity of Thea was higher in the meristematic tissues of the epiblast and the young shoot. By stage 4, the nutrients in the endosperm were almost depleted, at which time the Thea signals were at their highest in the epiblast, and the columnar sheaths of the shoot meristematic tissues contained a large amount of Thea but had not yet been transmitted to the bud. Interestingly, the Thea signal intensity was elevated in the apical root meristem during S4, whereas in the root elongation zone, Thea did not accumulate in the pericycle as in S2, but rather in the epidermal layer of the root maturation zone (Supplementary Data Fig. S5), indicating that at this time the synthesis of Thea is altered by the nitrogen source.

Glu is an essential key amino acid for plant growth and development, and it is also a major precursor substance for Thea synthesis. The distribution of Glu (*m*/*z* 148.0604, [M + H]^+^) in the four stages of tea seed cotyledons was exactly opposite to that of Thea. The signal intensity of Glu was low near the hypocotyl, and it was mainly enriched in the endosperm (Fig. 3c). The signal intensity of Glu in the root tip meristem during the S1–S4 periods was very weak. Its signaling peaks were mainly in the endosperm and the shoot at the S4 period (Fig. 3d), suggesting that it was involved in both protein degradation and sprouting, and had an important free ammonia regulatory role. Gln (*m*/*z* 147.0764, [M + H]^+^), another important amide compound, showed a similar distribution trend to Thea during the development of the radicle and germ (Supplementary Data Fig. S6), except that Gln was distributed in the endosperm in a small amount, but not synthesized during germ growth. According to the signal intensity distribution of Glu, Gln, and Thea in different organ development stages, most of the Glu is converted possibly into Thea and Gln to participate in nitrogen distribution and transport. Meanwhile, Thea and Gln are more involved in root growth and morphogenesis, and Glu plays an important role in the growth of tea buds.

Table 1 shows the content changes of other free amino acids, from which we selected some noteworthy amino acids for spatial distribution presentation (Supplementary Data Fig. S6). According to Supplementary Data Fig. S6a, Arg (*m*/*z* 175.1192, [M + H]^+^) was mainly distributed in the aleurone layers of the cotyledon. The Arg signal in the cotyledon during the S2 and S3 stages was significantly enhanced, indicating that a large amount of free Arg was produced by protein degradation at this time, but the distribution of Arg in the radicle and germ was not obvious (Supplementary Data Fig. S6b and c). By the S4 stage, Arg signal was reduced significantly in the cotyledon, and Arg was relatively high in the middle column area of the root, while Arg was concentrated around the pericycle in the stem meristem (Supplementary Data Fig. S6d). The distribution and variation of ornithine (Orn) (*m*/*z* 133.0975, [M + H]^+^) in different organs was similar to that of Arg. It is worth noting that although Orn content is not high and it is not a protein amino acid, obvious signal can be detected in endosperm and stlele , indicating that Orn is involved in the development of tea seeds. Phenylalanine (Phe) (*m*/*z* 166.0862, [M + H]^+^) had a great change in the cotyledon, which was similar to that of Glu. Phe distributed in the stem tips during the S3 stage, and there was no significant regional differentiation of stem buds in the S4 stage. The distribution of histidine (His) (*m*/*z* 156.0766, [M + H]^+^) exhibited more pronounced tissue specificity compared with the distribution of Phe and Orn (Supplementary Data Fig. S6c). According to Supplementary Data Fig. S6, His mainly concentrated in the endosperm region during S1–S3, and mainly distributed in the aleurone layer in the cotyledon during S4. The relative content of His in the whole root was high during S2, but it was more obviously enriched in the root cap in S4. In general, the content of His was relatively low in the epicotyl and bud. These results indicate that there are significant differences in the distribution of free amino acids during the growth and development of tea seedlings, contributing to tissue differentiation and organ formation.

### MALDI-TOF MS investigation of starch metabolism and glycolysis products

**Figure 4:**
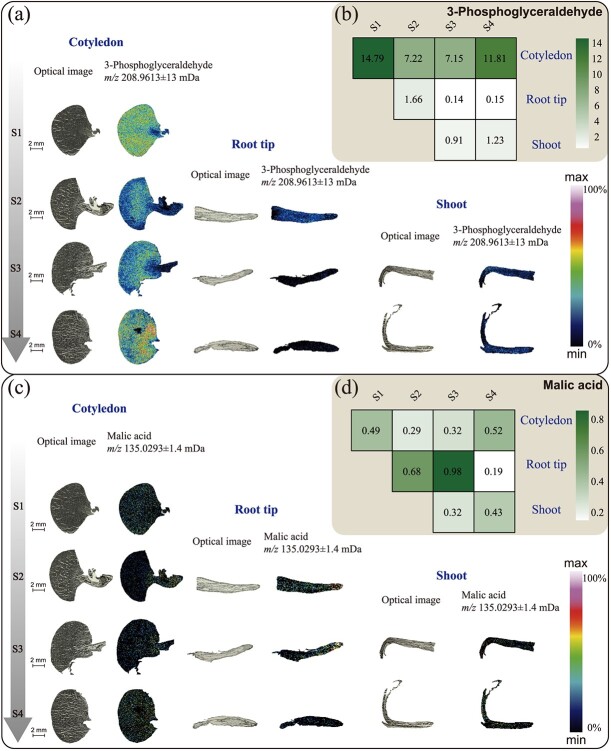
Spatial MSI distribution and relative contents of 3-phosphoglyceric acid and malic acid. **a**, **c** Optical images (left) and ion distribution images (right) of 3-phosphoglyceric acid and malic acid in cotyledons, root tips, and stems (leaf buds) at four developmental periods. The color scale indicates increasing content of target substances from dark black to purple in a sequential manner in the region. **b**, **d** Heat maps of the relative content of 3-phosphoglyceric acid and malic acid in different samples. The color scale indicates a sequential increase in relative content from white to green.

In this study, the contents of glucose-6-phosphate (isomer mixture), glyceraldehyde 3-phosphate, phosphoenolpyruvate, oxaloacetic acid, citric acid (isomer mixture), malic acid, succinic acid, dextrin, lactose, galactose (glucose), raffinose, and a small number of carbohydrate complexes were detected during glycolysis and the TCA cycle. As shown in Fig. 4 and Supplementary Data Fig. S7, almost all of these intermediate metabolites in the TCA cycle and glycolysis during the tea seedling germination process were highly accumulated in the cotyledons and embryos, which was related to starch degradation and glycolysis during the germination process. As the tea seeds grew and developed (S1–S4), these intermediate products gradually transferred from cotyledons to embryos. As shown in Supplementary Data Fig. S7, dextrin (*m*/*z* 543.1323, [M + K]^+^) was detected in the cotyledons during various stages, but the developing embryos at each stage contained higher levels of dextrin than other parts. During the S2 stage, the dextrin signal in the root was mainly detected in the central column of the apical meristem, while during stages S3–S4 the signal intensity in the apical meristem significantly decreased and was evenly distributed. During the S3 and S4 stages, the dextrin signals were highly concentrated in the stem meristem and elongation regions, but the signals from the top buds and new shoots were relatively weak. Interestingly, dextrin signals were stronger in the hypocotyl and root mature cortex than in the middle column during S4 stage. Raffinose (*m*/*z* 527.1581, [M + Na]^+^) and d-galactose (glucose) (*m*/*z* 203.0529, [M + Na]^+^) were hardly detected in the endosperm during the four periods, and they were present only in small amounts in the dextrin layer, seed coat, and embryo axis. The detectable signals of raffinose and d-galactose (glucose) in the root tip meristematic zone were very strong and almost evenly distributed. With the development of tea seedlings, the signals of both were detected in the apical bud, stem meristem, and elongation area (S3). Interestingly, raffinose was less distributed in the stem mature area, while d-galactose (glucose) was enriched in the stem mature area. During the S4 period, raffinose and d-galactose (glucose) had strong signals in the middle column sheath of the stem meristem and elongation regions. The signals of raffinose and d-galactose (glucose) were stronger in the cortex of the root mature area and the cortex of the upper and lower embryonic axes during the S4 period, but the d-galactose (glucose) signal was almost absent in the pericycle during the S4 period. Glucose-6-phosphate, glucose-1-phosphate, and fructose-6-phosphate were not separated in this detection for isomeric reasons, so the total amount of the three was investigated (Supplementary Data Fig. S7). During the four stages, glucose-6-phosphate, glucose-1-phosphate, and fructose-6-phosphate were evenly distributed in the cotyledons, root tips, and stems. In the S2 stage, the only strong signal was detected near the hypocotyl, while in the S4 stage strong signals were detected in the upper and lower hypocotyls and were concentrated in the central column. However, in the mature zone of the S4 root tissue, the central column was slightly lower than the cortex. In general, starch metabolism has obvious tissue specificity during tea seedling development, and the distribution of glycolysis products is relatively uniform.

**Figure 5:**
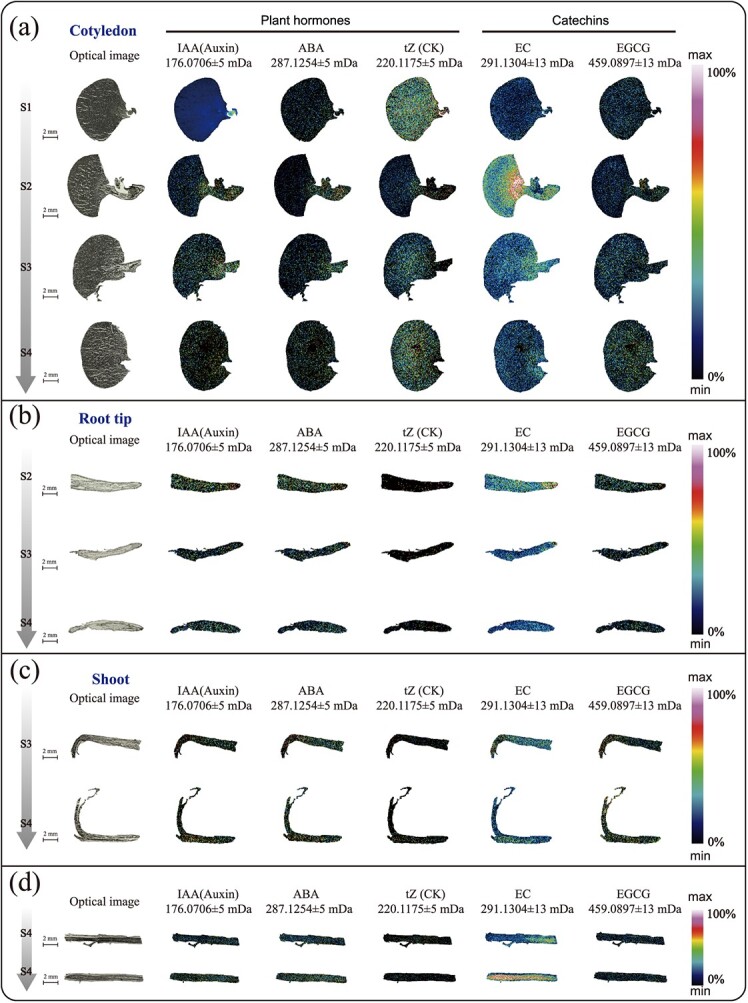
Spatial MSI distribution results of IAA (indole-3-acetic acid), ABA (abscisic acid), tZ (*trans*-zeatin), EC (l-epicatechin), and EGCG (epigallocatechin gallate) in different tissue samples. The leftmost is the optical image; others are ion distribution images. Cotyledon (**a**), root tip (**b**), stem (leaf bud) (**c**) and embryonic axis (left and right) (**d**) at S4 period and mid-section of radicles at S4 period.

As shown in Fig. 4a and b, 3-phosphoglyceraldehyde (*m*/*z* 208.9613, [M + K]^+^) was mainly detected in the cotyledons during the four stages. Its relative content was highest in S1, and decreased in stages S2 and S3. In addition, 3-phosphoglyceraldehyde was also distributed to a small extent in the root apical meristem and the shoot. Malic acid (*m*/*z* 135.0293, [M + H]^+^) was present in the cotyledons, root tip meristems, and stem buds of tea seedlings during growth and development; its relative content increased in the vicinity of root crowns during stages S2 and S3, and suddenly decreased in the root tip region during stage S4, when the detectable signal was very weak. The signals of oxobutanedioic acid (*m*/*z* 133.0157, [M + H]^+^), succinic acid (*m/z* 141.0172, [M + Na]^+^), and the isomer mixture of citric acid and isocitrate (*m*/*z* 193.036, [M + H]^+^) were very weak in the cotyledons during the four stages (Supplementary Data Fig. S8). Succinic acid had no obvious tissue distribution and weak signals in various stages and parts. Oxaloacetic acid was evenly distributed and its content was relatively high in the root tip during S2, but significantly decreased in the root tip region during S3 and S4. The distribution of oxaloacetic acid was higher in the stem tip and cortex compared with other stem regions during S3, but decreased and was evenly distributed during S4. The signal intensity of oxaloacetic acid was slightly higher in the cortex of the root maturation region and the cortex of the upper and lower embryo during S4 than in the mid-column region. The signal intensity of citric acid and isocitric acid mixture in the root cap was lower than that in the root tip meristem at three stages (S2–S4). With root development, the signal intensity in the root tip area gradually increased during stages S3 and S4, and during the S4 period the two were mainly distributed near the cortex of the mature root zone and the cortex of the upper and lower hypocotyls. In the shoot, the mixture of citric acid and isocitric acid was mainly distributed in the apical bud and cortex during stage S3, while it was mainly distributed in the new shoots at stage S4. These results showed that oxaloacetic acid, malic acid, citric acid, and isocitric acid mainly changed in the root tips and new shoots during the TCA cycle.

### MALDI-TOF MS investigation of other metabolites in different tea seedling tissues during different developmental stages

This study also found some metabolites, including auxins, cytokinins (CK), abscisic acid (ABA), and the characteristic secondary metabolites of tea tree, epigallocatechin and epigallocatechin gallate, which may be related to the growth and development of tea seedlings. Auxins, ABA, and cytokinins were distributed in all parts of the tea seedlings during the four periods (Fig. 5). In the S1 stage IAA was abundant in the hypocotyl and germ, but the contents of cytokinin and ABA were relatively low. With the growth and development of tea tree seedlings, the expression of auxins and ABA in tea seeds basically showed the same trend, gradually increasing from S1 to S3, but the contents of both began to decrease in the S4 stage (Fig. 5a). ABA did not show significant regional specificity in the cotyledons of the S1 stage, while it was highly enriched in the hypocotyls of S2 and S3 stages (Fig. 5a). In addition, auxin and ABA showed no obvious tissue specificity during apical development, but the hormone content was lower in the mature roots of S4 and near the stele of the upper and lower embryonic axis than in the cortex (Fig. 5d). The distribution of cytokinins in the root tip and stem was generally low, and the cytokinins were concentrated in the pericycle region (Fig. 5c). Although the hormone distribution in the parts tested during all stages had no obvious tissue specificity and the signal was weak, plant hormones were distributed in all parts of the plant, indicating that plant hormones had important functions in all tissues during the growth of tea tree seedlings.

The distribution of epicatechins (ECs) (*m*/*z* 291.1304, [M + H]^+^) in tea seeds during the S1–S4 periods showed a trend of first increasing and then decreasing (Fig. 5). There was no significant tissue specificity in tea seeds during the S1 and S4 periods, but EC was mainly enriched in the embryo during the S2 and S3 periods. During the S2 and S3 stages, EC was mainly enriched in the root cap, while during the S4 stage there was no significant tissue specificity in the root tip meristem (Fig. 5b). The relative EC contents of the stem and shoots during the S3 stage and new shoots during the S4 stage were relatively high, and the EC content of the middle column in the stem was slightly higher than that in the cortex and other parts. During the S4 period, the hypocotyl and mature zone roots mainly existed in the root stele. Combined with the EC enrichment in the stem, there was a trend for the transfer of catechins from the stem to the stem cortex during the S4 period. Epigallocatechin gallate (EGCG; *m*/*z* 459.0875, [M + H]^+^) exhibited no significant tissue-specific distribution, but the signal detected in the new shoots was strong. These results indicate that catechins are distributed in and exhibit a certain trend of change in various tissues during the development of tea seedlings.

## Discussion

Seed germination is a complex process that involves a series of morphological and physiological changes [31, 33–35]. In order to better demonstrate the process of tea seed germination and seedling establishment, samples from different organs were collected in five stages, SE–S5 (Supplementary Data Fig. S2), and the spatial metabolome was used to analyze the physiological changes based on the continuous gradual changes in the metabolite hierarchy. In this study, the spatial distribution of amino acids, sugars, organic acids, polyphenols, and hormones during the growth and development of tea seedlings was clearly mapped using the MALDI-MSI method, and their distribution changes in different tissues at different developmental stages were investigated, which provides a new perspective for understanding the nutritional and metabolic characteristics of tea seeds during development. The results indicated that nitrogen metabolism was dominated by Thea, while carbon metabolism was dominated by glycolysis and the TCA cycle, and revealed the possible effects of some hormones, polyphenols, and other substances on the growth and development of tea seedlings.

### Theanine is strikingly synthesized during tea seed germination, meaning that it plays the most important role of nitrogen metabolism node

During the imbibition and germination stage of seeds, the growth of the embryo first utilizes soluble sugars, amino acids, and only a small amount of storage proteins from the embryo or embryonic axis. Subsequently, the storage substances in the endosperm begin to decompose into free amino acids and monosaccharides, which are transported to the embryo. A portion of these substances serves as a respiratory substrate, while the other portion is synthesized at the growth site to form materials for new cells [36, 37].

During the early stage of tea seedling development, the crude protein accumulated in the endosperm is mainly hydrolyzed to provide free amino acids for the tea tree. This study revealed a special nitrogen metabolism phenomenon during tea seedling development, in which the hydrolyzed protein in the endosperm is synthesized in large quantities into Thea in the embryo and transported to various tender organs. From Table 1, it can be seen that Thea contributes the largest proportion of amino acids in the roots, stems, and leaves during the development of tea tree seedlings. This indicates that nitrogen transport and redistribution are mainly conducted in the form of Thea during the development of tea seedling. Previous studies have shown that Thea is biosynthesized by glutamate and ethylamine via Thea synthase (CsTSI) [1, 38]. As Thea has a similar chemical structure to Gln, it is generally believed that Thea has similar biological functions to Gln, such as providing a source of nitrogen. However, this study showed that the relative contents of amino acids involved in nitrogen storage, such as Gln, Asp, and Arg, in various organs during tea seed development were similar to those of other plants, indicating that these essential amino acids for protein composition were involved in various processes of tea seed development, and Thea, as a non-protein amino acid, was synthesized in a large amount during the germination of the tea seed embryo. In addition to nitrogen transport and redistribution, does Thea have other regulatory functions?

### Theanine may be involved as a nutrient signaling molecule in the early stages of tea seedling development

**Figure 6:**
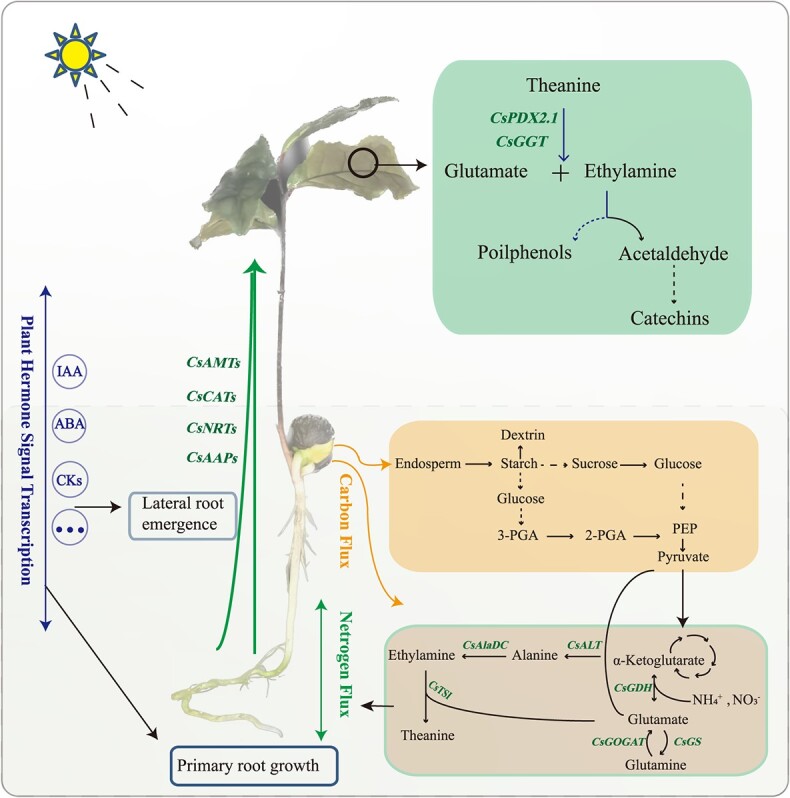
Diagram of carbon and nitrogen metabolism and hormone signaling patterns during the development of tea seedling.

The investigation of Thea and its anabolic-related products using MALDI-MSI revealed that the initiation of Thea biosynthesis was a very early event in the germination and growth of tea seedlings. As it is difficult to detect Thea and ethylamine in ungerminated tea seeds, the results of spatial metabolomics showed that Thea was first synthesized during the imbibition period of tea seeds, and was rapidly synthesized during the radicle germination stage (Fig. 3a), with obvious tissue specificity, mainly in the embryo. However, the radicle is still the site of a large amount of Thea synthesis and accumulation, and Thea is very likely to be synthesized in the pericycle of the root, which may be related to the location of *CsTSI* in the pericycle [38, 39]. Subsequently, some transporters (such as CsAAPs and CsCATs ) transport Thea from the columnar sheath in the meristem zone to the cortex on both sides of the root mature zone [8, 40], appearing in the lateral roots during the S4 stage (Supplementary Data Fig. S4). Early research found that the Thea content in the lateral roots of tea seedlings was higher than that in the main roots [38]. Chen *et al*. found that the exogenous application of Thea could regulate lateral root development [41]. In addition, lateral root development originates from the central column of the primary root [42]. Therefore, we suspect that the transfer of Thea from the pericycle of the meristematic zone to the cortex on both sides of the mature zone may occur to stimulate the development of lateral roots and increase the absorption of nutrients. The present study found that the Thea content in each young organ was the highest at the early stage of development, and then gradually decreased. According to the distribution of Thea in different organs revealed by the spatial metabolome, Thea is mainly synthesized in the meristem zone of the root apex and transported in the elongation zone and mature zone. However, in the region with the most vigorous organ differentiation, because Thea is not the amino acid that makes up the protein, Thea may be decomposed into Glu under the action of some Thea hydrolases to participate in tissue formation (Fig. 3c). For example, CsPDX2.1 and CsGGT2 have been reported to hydrolyze Thea in the leaves (Fig. 6) [42, 43].

In the early autotrophic stage of growth and development, tea seedlings provide energy and substrate mainly through the catabolism of endosperm proteins in cotyledons. Subsequently, the nitrogen nutrients, mainly theanine, flows to the aboveground leaves for catabolism and participation in organ formation through transporter proteins, which not only provides nitrogen nutrients for growth and development to maintain the carbon and nitrogen balance, but also has the potential to act as a signaling molecule to regulate the growth and development of the tea seedling. At the same time, phytohormone signaling in this process may regulate tea seed germination, embryonic root elongation, lateral root genesis and aboveground development of the tea seedling.

Thea first appeared in the embryo with the strongest meristematic activity at the radicle germination stage (S1) of tea seeds. Thea was concentrated in the root elongation zone at the radicle developmental stage (S2), but the Thea content of the root tip was extremely low. During the S3 and S4 stages, as the aboveground parts begin to grow and develop, Thea was concentrated in the stem and leaf differentiation area, but its content was extremely low in the leaf primordia (Fig. 3a). This means that Thea may be synthesized or transported to young organs, but it cannot directly participate in the differentiation of new tissues and cell morphology construction. In this series of processes, the synthesis, transport, and transformation of Thea are regulated by complex signals. As the most important nitrogen form, we speculate that Thea may also act as a nutrient signaling molecule to regulate the development of tea seedlings, and these results are also consistent with previous speculations [12].

### Sugar metabolism and TCA cycle products provide energy and substrates for the early stages of tea seedling development

At the initial stage of germination (SE), the seeds mainly generate glucose 1-phosphate (G-1-P) through starch phosphorylation, and then glycolysis occurs to provide energy. At the later stages of germination (S1, S2), α-amylase and β-amylase activity increased, and hydrolysis became the main route of starch degradation to support the development of seedlings until the aboveground part grew for photosynthesis (Fig. 6) [44]. The starch hydrolysis process is mainly induced by the production of gibberellin in the coleoptile, which enters the aleurone layer and induces cell production. The α-amylase enters the endosperm for hydrolysis, and its products are transported to the growing embryo [44, 45]. This is consistent with the results of dextrin content distribution illustrated in Supplementary Data Fig. S7. It is worth noting that from the distribution of carbohydrate substances detected using the spatial metabolome, dextrin not only had a strong signal in the cotyledons, but also had a clear distribution in the roots, stems, and leaves of other developmental stages. This indicates that starch decomposition products not only provide the necessary materials and energy for carbon metabolism in the form of G-1-P, but they also may participate in the development of new organs and tissues through the direct transport of dextrin [46, 47]. In addition to starch, fat is also an important storage substance in seeds. The fat in cell liposomes is first hydrolyzed into fatty acids and glycerol by fat hydrolase. Fatty acids are catabolized by the β-oxidation process; the esteroyl coenzyme A is oxidized to produce succinic acid through the glyoxylate cycle, and then oxaloacetic acid is generated through the citric acid cycle, and can be partially converted into sucrose through gluconegenesis and transported to the growth site. The results of spatial metabolomics showed that the content of oxaloacetic acid directly generated by fatty acid metabolism in the cotyledons was extremely low. Oxaloacetic acid was mainly transformed in the hypocotyls and transported to the roots and buds (Supplementary Data Fig. S7). Another product, glycerol, is rapidly phosphorylated in the cytoplasm to form glycerol 3-phosphate, which is then dehydrogenated to form dihydroxyacetone phosphate via glycolysis to produce energy, As can be seen from Fig. 4a and b, the synthesis of glyceraldehyde 3-phosphate in the endosperm was greatly activated and subsequently transported to the radicle and germ to participate in energy metabolism, indicating that phosphorylation in the endosperm was more active during the germination of tea tree seedlings.

Glycolysis in the plastids and cytoplasm and the mitochondrial TCA cycle are major components of glucose metabolism and respiratory metabolism, providing carbon and energy to cells. This study found that the intermediate products of the glycolysis process were mainly distributed in the growing parts of the tea seedlings, such as the hypocotyls, root tips, stem tips, and new shoots, providing substrate and energy for the synthesis of tissues and cells. The dextrins and phosphorylated monosaccharides generated during carbon metabolism were the most widely distributed and were present in all organs, providing a basis for the formation of carbon skeletons. Interestingly, d-galactose (glucose) accumulated extensively in the late stages of root development, suggesting that it may be involved in the formation of arabinogalactin proteins, thereby affecting root development.

### Possible roles of other related compounds in tea seedling development

Plant hormones are widely present small molecules produced by plant metabolism and are important signaling compounds. They are key regulatory factors for seed germination, root, stem, and leaf development, flowering, biotic and abiotic stress responses, and many other biological activities [48, 49]. The three hormones found in this study were distributed in various tissues during the growth and developmental stages of tea seedlings. Although the signal is generally not high, the function of hormones in the development process of tea seedlings is indispensable. Studies have shown that ABA, auxins, and cytokinins are involved in nitrogen signaling and root development [50]. Nitrate signaling controls plant growth and development through signaling pathways involving plant hormones [51]. However, cytokinin signaling is weaker than that of auxins and ABA in root tip development, at least according to the current results, and auxins and ABA may be more important in root tip development. Catechins have been proposed to play an important role in the anti-stress regulation of secondary metabolic pathways, and they have a vital role in the resistance of tea plants to pathogens [52]. However, there was almost no obvious tissue distribution of catechins throughout the entire developmental period investigated in this study, except in the root mature region, so it may be valuable to study the distribution of catechins in the root mature region.

In summary, the growth and development of tea tree seedlings is the result of the combined action of nitrogen metabolism dominated by Thea, glycolysis, TCA cycle metabolism, phytohormones, and other substances. The spatial distribution of these metabolites during the growth and development of tea seedlings may provide a reference for the study of the role of these substances themselves and for tea tree breeding.

## Materials and methods

### Plant materials

Tea seedlings were produced from fresh tea seeds of the variety ‘Shucha Early’ in Shucheng County, Anhui Province, China, and were cultivated and sampled in the greenhouse (greenhouse conditions: light duration of 12 h/day, temperature of 22 ± 1°C, light intensity of ~270 μmol m^−2^ s^−1^, air humidity of 35–40%) of Anhui Agricultural University’s Nongcui Garden. Tea seeds were sown in a mixed soil of nutrient soil:vermiculite:perlite in a ratio of 1:2:1. Samples were collected from various parts of tea seedlings at different developmental stages, as shown in Fig. 1a. The samples were collected from the following five stages: SE, seeds; S1, sprouting and revealing the growing point of the germ (~10 days); S2, germ growing (~20 days); S3, growing young stems (~30 days); S4, one bud growing, with two or three leaves (~40 days). After sample collection was completed, the samples were divided according to organs, and a portion of each sample was tested for amino acid content and ethylamine content, and each sample was set with three biological replicates. The other part was cut to a suitable size with a scalpel, placed in an embedding box (the box cover marked the direction of the sample), and completely embedded with 2% (m/v) carboxymethyl cellulose solution. The embedded samples were then frozen in a dry ice–ethanol bath. When the embedding agent had become completely white, the embedding box was removed, stored in a refrigerator at −80°C, and reserved for spatial metabolomics detection. Section directions of different organs during the development of tea seed seedlings are shown in Supplementary Data Fig. S2.

### Determination of free amino acid content

Referring to the method of Huang *et al*. [53], a fully automated amino acid analyzer (L-8900, Hitachi, Japan) was used for detection. The main free amino acid extraction steps were as follows: (i) 0.1 g of lyophilized sample was weighed and 4% sulfosalicylic acid was added to a total volume of 10 ml, followed by ultrasonic extraction for 30 min, during which the sample was inverted and stirred every 5 min; (ii) after ultrasonication, the sample was allowed to stand for 10 min, and the supernatant was taken and centrifuged for 30 min at 12 000 rpm; and (iii) after centrifugation, the supernatant was taken out and then passed through the a 0.22-μm disposable aqueous membrane for amino acid analysis.

### Determination of ethylamine content

The method used for the detection of ethylamine in tea referred to previous literature [43]. As ethylamine is a volatile substance, GC–MS (7890B-5977B, Agilent Technologies Inc., USA) was used for detection. Samples were processed as follows: (i) 25 mg of tea tree sample (lyophilized sample) was taken and 1.5 ml of Watson’s water was added; (ii) after a water bath at 85°C for 5 min and standing at room temperature (25°C) for 2–3 h, the supernatant was collected by centrifugation at 3500 g; (iii) 2 ml was placed in a silanized Eppendorf tube and 500 μl of toluene was added, followed by 500 μl of supernatant; (iv) 400 μl of phosphate buffer was added; (v) 25 μl of isobutyl chloroformate solution was added; (vi) 5 μl of deuterated internal standard, ethylamine hydrochloride, was added to a final concentration of 2 mg/ml; (vii) the solution was centrifuged at 250 rpm for 10 min; (viii) the solution was centrifuged at 3500 g for 5 min; (9) 200 μl of toluene supernatant was collected and placed in a dedicated 1.5-ml centrifuge tube; (x) 200 μl of potassium hydroxide saturated methanol solution was added and shaken for 5 min; (xi) 600 μl of 5 mol/l NaOH solution was added; (xii) the solution was centrifuged at 250 rpm and 3500 g for 5 minutes respectively; and (xiii) the upper toluene solution was passed through a 0.22-μm organic phase membrane into a liquid phase vial for subsequent detection.

### MALDI sample preparation

The sample preparation and pretreatment processes for MALDI–MSI samples were modified slightly based on the methods described in published papers [26, 54]. The samples frozen in the embedding material were removed and placed in a microtome at −20°C for 1 h to equilibrate. Subsequently, the tissue was automatically sliced using a cryoslicer (CM1950, Leica, Germany), with a slice thickness of 30 μm. The cut section was transferred to a pre-cooled indium tin oxide (ITO) slide using a pre-cooled brush, the back of the slide was placed against the back of the researcher’s hand, and the temperature of the back of the hand was used to melt the tissue section until it became transparent. After the section became transparent, the tissue turned from transparent to white when the back of the slide was rubbed with the fingers and the water on the front side of the slide evaporated due to the temperature of the back side. After cutting, the ITO slides containing tissue sections were dried under vacuum for 30 min.

A matrix solution of 15 mg/ml 2,5-dihydroxybenzoic acid (DHB) dissolved in 90% acetonitrile was uniformly sprayed onto ITO slides containing tissue sections using a TM-Sprayer matrix sprayer for a total of 28 cycles, with a drying time of 5 s between cycles, temperature 60°C, flow rate 0.12 ml/min, and pressure 6 psi.

### Image detection and mass spectrometry results

Image detection and MS were carried out as described by Xia *et al*. [29] and Liao *et al*. [30] and were divided into the following main areas. The ITO glass slide with the sprayed matrix was placed on the target plate of the mass spectrometer (Bruker Daltonics, Bremen, Germany), and the organization area was selected through Data Imaging (Bruker) software. The imaging area was evenly divided into 2D dot arrays consisting of a number of dots according to their size. The MS signal was detected in the mass range of *m*/*z* 50–1300 Da in positive ion mode. Under the same laser energy, tissue samples were detected. The laser beam irradiated the tissue region on the target plate through the grating, and the samples were continuously scanned. Individual tissue samples from tea seedlings were subjected to a laser beam for matrix ionization analysis, while the released molecular signals were captured and identified using a mass spectrometer to obtain information on the mass-to-charge ratio (*m*/*z*) of each tissue spatial region in the sample and raw peak intensity area data (raw data). The raw data were read by importing SCiLS Lab software, then smoothed and root mean square normalized to obtain the *m*/*z* information of each spatial point and transformed into pixel points on the imaging thermogram. The target peak with higher intensity (MS first-level information) was *in situ* splintered on the tissue to obtain the second-level atlas (MSMS second-level debris peak information) collected on the tissue. Subsequently, substance identification was carried out using the collected secondary spectra by searching the self-built database integrated with the public database (HMDB, https://hmdb.ca/). Low-intensity substances for which secondary target peaks (MS first-level information) could not be collected were searched by their first-level molecular weights (within 10 ppm of the error range) in the self-built database integrated with the public database (HMDB) to identify the substance closest to the molecular weight detected by the instrument. The relative quantitative value of each substance in each tissue in different regions was expressed by the peak intensity value of the target peak after root mean square normalization. The higher the value, the higher the content of the target object in this region.

### RNA extraction and real-time quantitative PCR

Total RNA extraction, cDNA preparation, and quantitative real-time PCR (qRT–PCR) methods were consistent with previous descriptions [43]. Briefly, qRT–PCR analysis was performed using Bio-Rad software according to the ChamQ Universal SYBR qPCR Master Mix (Vazyme, China) method, with CsGAPDH as an internal reference and qRT–PCR amplification using gene-specific primers (Supplementary Data Table S3). The cDNA synthesized from three independent RNA extracts was set up as replicates and analyzed for quantification using the 2^−ΔCq^ method.

### Data processing and statistical analysis

Data and image processing refer to the published literature [54]. In this study, SPSS 21.0 software was used for statistical processing of the data. The mean and standard error of the three bioreplicates were calculated. LSD and Duncan’s multiple range tests were performed after one-way ANOVA. *P* < 0.05 was considered statistically significant. PCA diagrams of different tissue samples of tea seedlings were drawn using SIMCA 14.1 (Umetrics, Sweden). Heat maps were drawn using software R 4.2.2.

## Supplementary Material

Web_Material_uhae218

## Data Availability

All relevant data are available within the article and its supplementary data.
