# Supplementary material for: Visualization of metabolite distribution based on matrix-assisted laser desorption/ionization–mass spectrometry imaging of tea seedlings (Camellia sinensis)
Source: Hortic Res. 2024 Aug 3;11(10):uhae218. doi: 10.1093/hr/uhae218 (PMC11469920; doi:10.1093/hr/uhae218)
Supplement: Web_Material_uhae218 [file web_material_uhae218.zip › Supplementary Figure.docx]

**
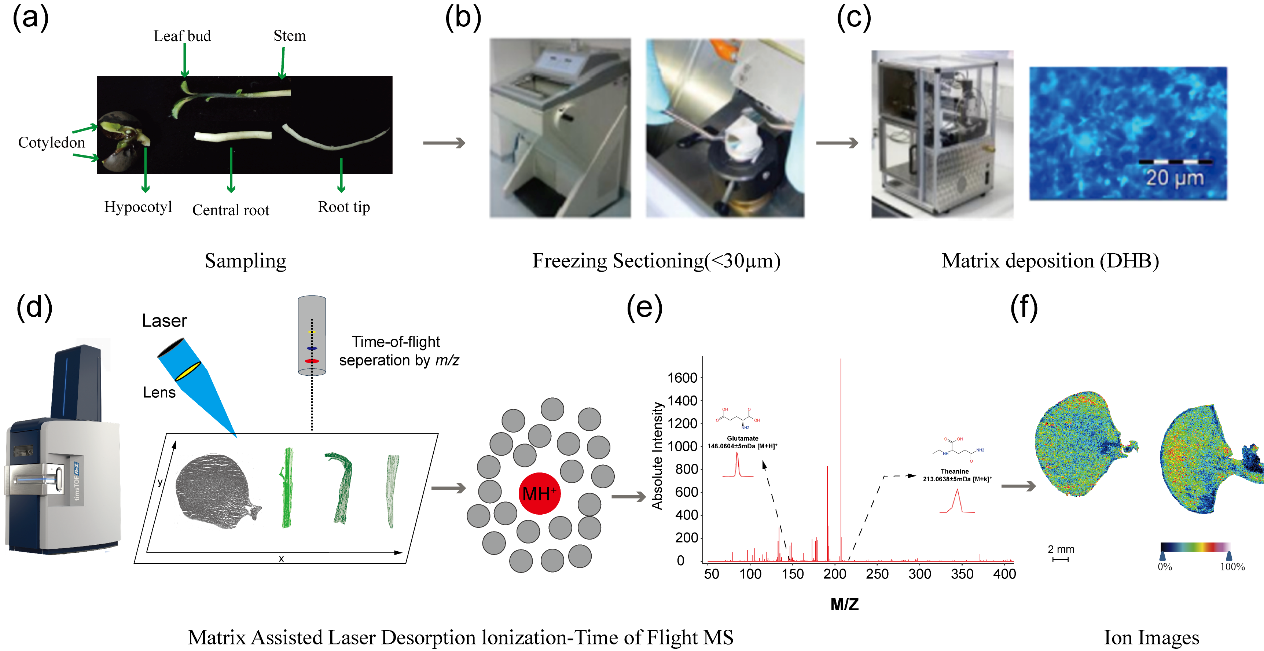
**

**Figure S1**. Flowchart of spatial mass spectrometry detection for the different tissues of tea seedling at different developmental stages. (a) Tea seedling tissue samples for MALDI-MS analysis are shown in detail. (b-f) Experimental flow of sample collection, preparation and mass spectrometric imaging. The samples were first cryosectioned with a slice thickness of less than 30 µm, followed by substrate spraying of the slices with DHB, and finally detected using MALDI-MS.

**
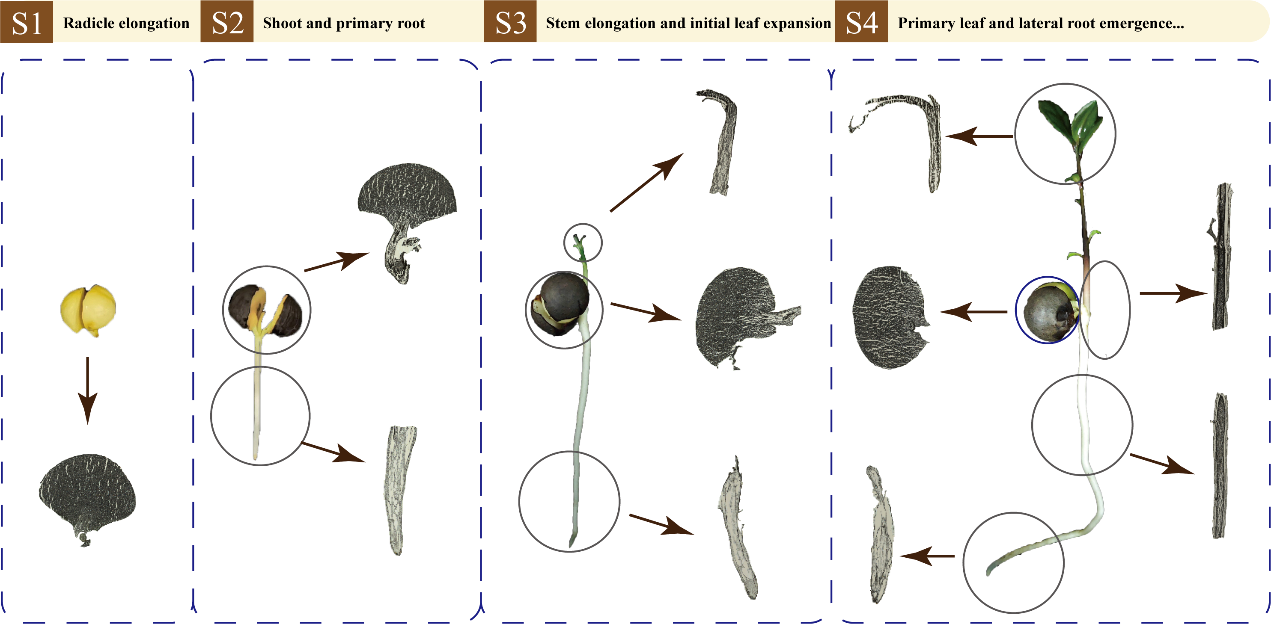
**

**Figure S2**. Schematic diagram of longitudinal sections of various organs of tea seedling in S1-S4 period.

**
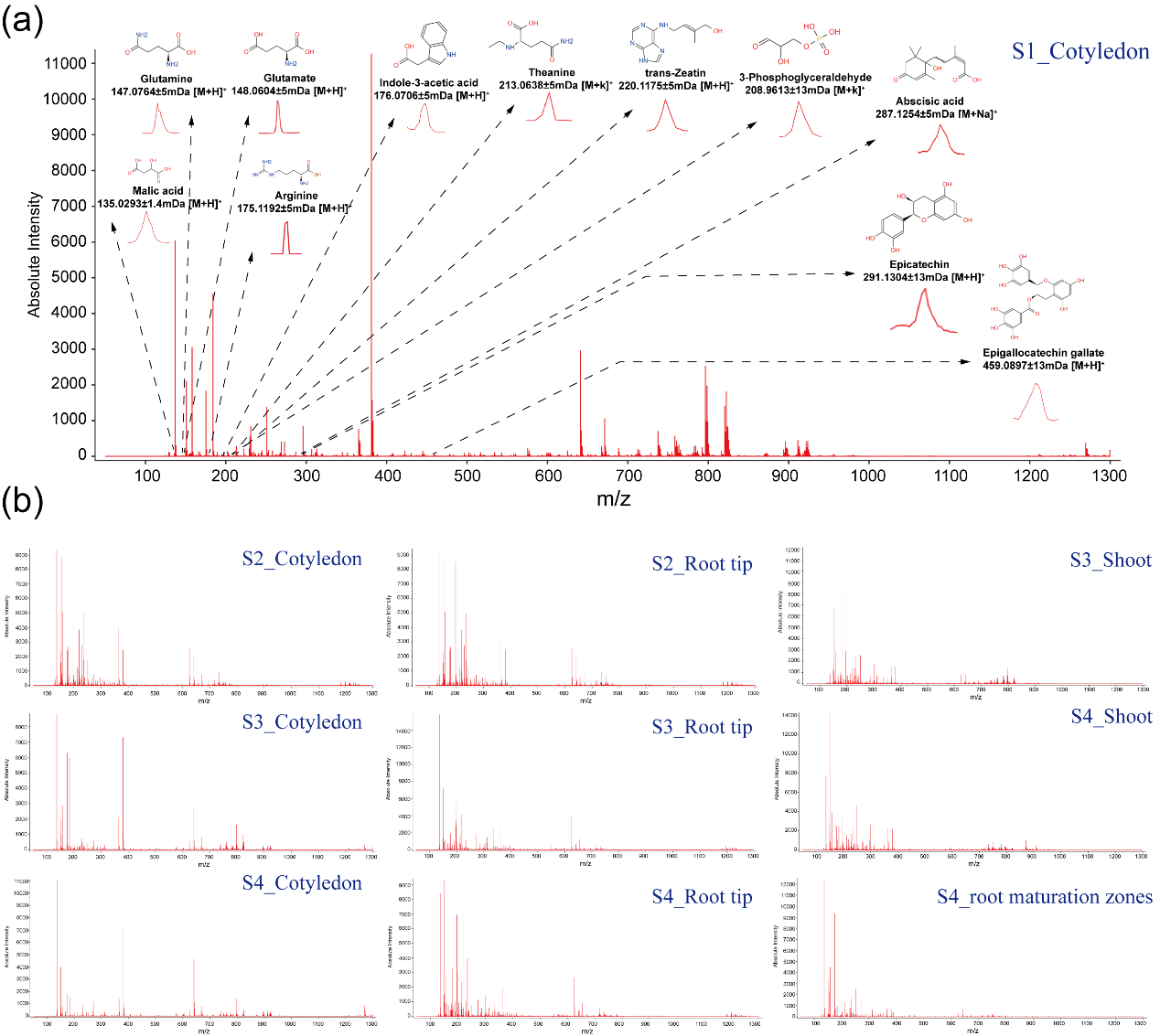
**

**Figure S3**. MALDI averaged mass spectra in the m/z 50-1300 mass range were obtained in the positive ion mode for different stages of the tea seedling development. (a) Some identified compounds in the cotyledons during the S1 period labeled with m/ z and compound name. (b) Average mass spectra of the other tissue sites. For more detailed information on the identification of other compounds, see supplementary table 1 and table 2.

**
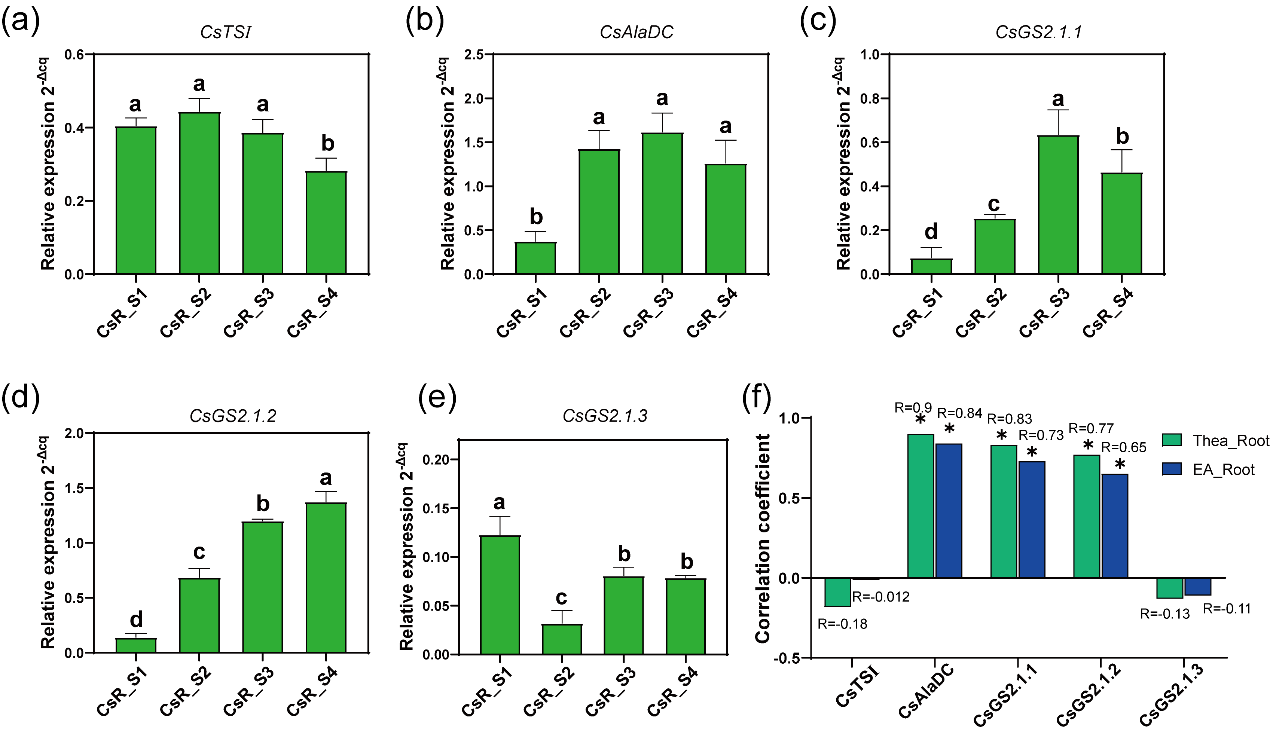
**

**Figure S4**. (a), (b), (c), (d), (e) indicate the expression of *CsTSⅠ*, *CsAlaDC*, *CsGS2.1.1*, *CsGS2.1.2*, and *CsGS2.1.3* in tea seedling. The internal standard was CsGAPDH. All data are expressed as mean ± SD (n = 3), p < 0.05 is indicated by different letters, and Duncan's Multiple Extreme Difference Test was used. (f) Correlation coefficients between the expressions of the theanine biosynthesis related genes with the theanine content. Asterisks indicate significance by Duncan's multiple extreme variance test (* *p* < 0.05).

**
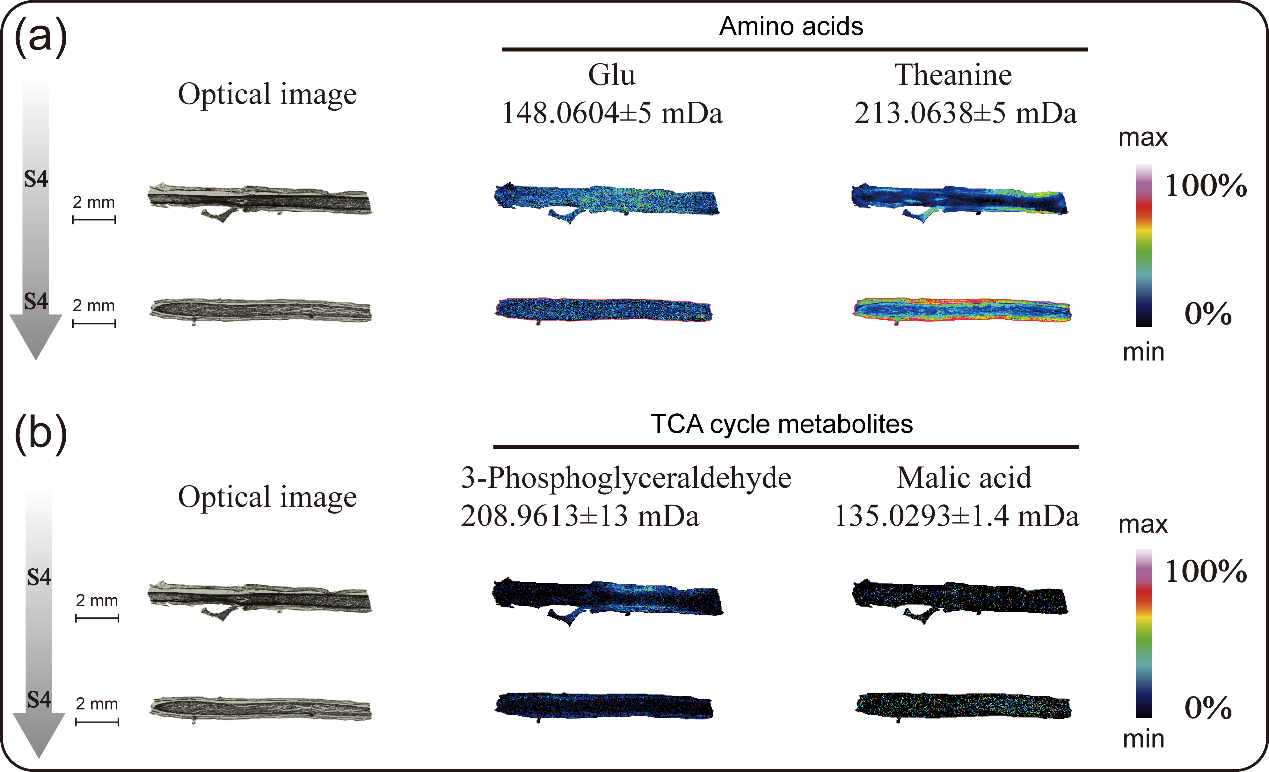
**

**Figure S5**. Spatial mass spectral distribution results of some compounds in the middle of epicotyl and hypocotyl (up) and root maturation zones (down) during S4. Optical images (left) and ion distribution images (right) of glutamate, theanine, 3-phosphoglyceraldehyde and malic acid, with color scales ranging from blue to red, representing the sequential increase of the target substances in the region.


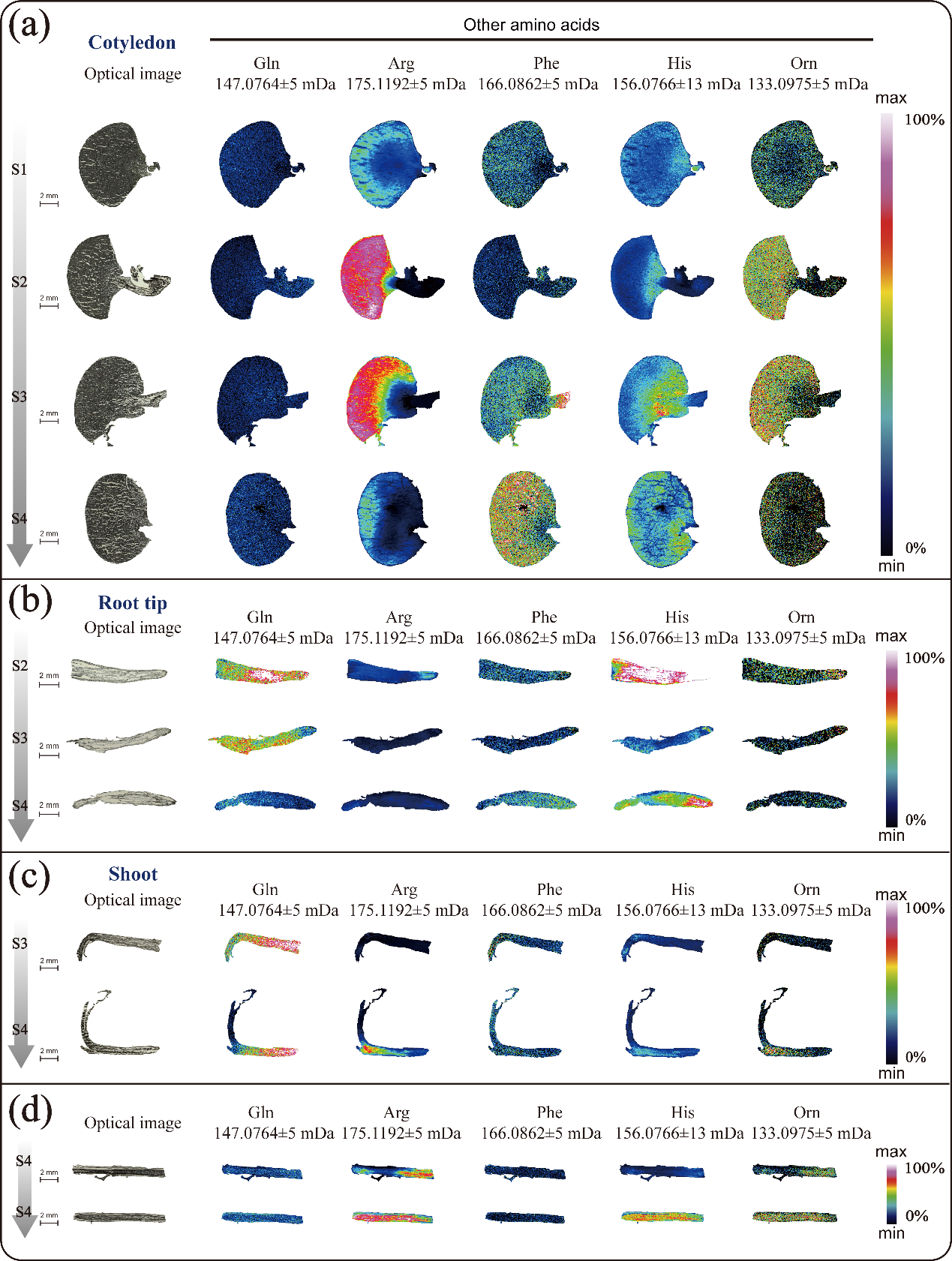


**Figure S6**. Spatial mass spectral distribution of some different amino acids. Optical images (left) and ion distribution images (right) of Gln, Arg, Phe, His, and Orn in the cotyledons(a), root tip(b), epicotyl(c), and the middle of epicotyl and hypocotyl (up) and root maturation zones (down) (d) at different stages. The color scale goes from blue to red, representing a sequential increase in the content of the target substance in the region.


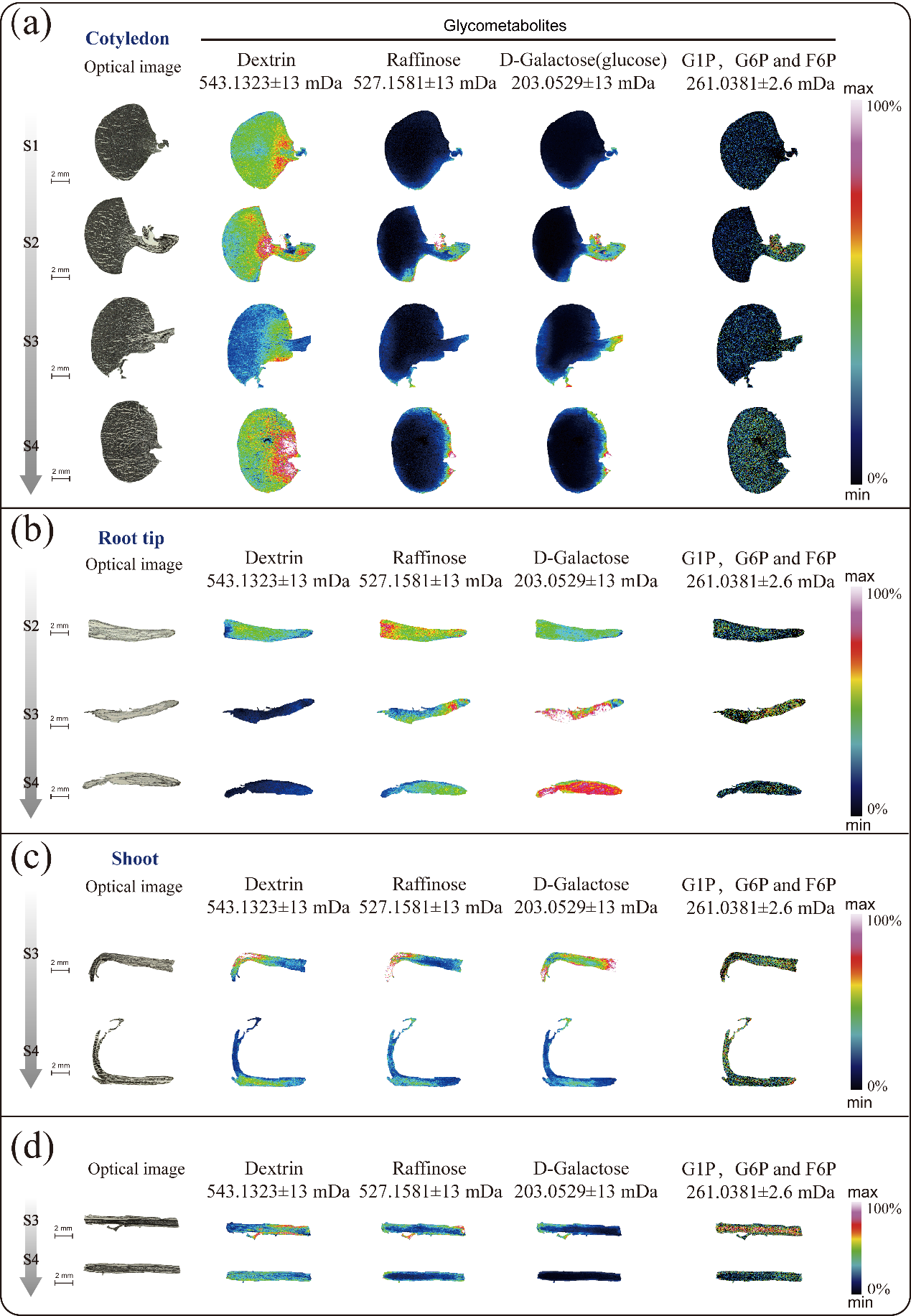


**Figure S7**. Spatial mass spectral distribution results of some glycol-metabolites. Optical images (left) and ion distribution images (right) of dextrin, raffinose, D-galactose (glucose), G1P, G6P, and F6P in the cotyledons(a), root tip(b), epicotyl(c), and the middle of epicotyl and hypocotyl (up) and root maturation zones (down) (d) at different stages. The color scale goes from blue to red, representing a sequential increase in the content of the target substance in the region.


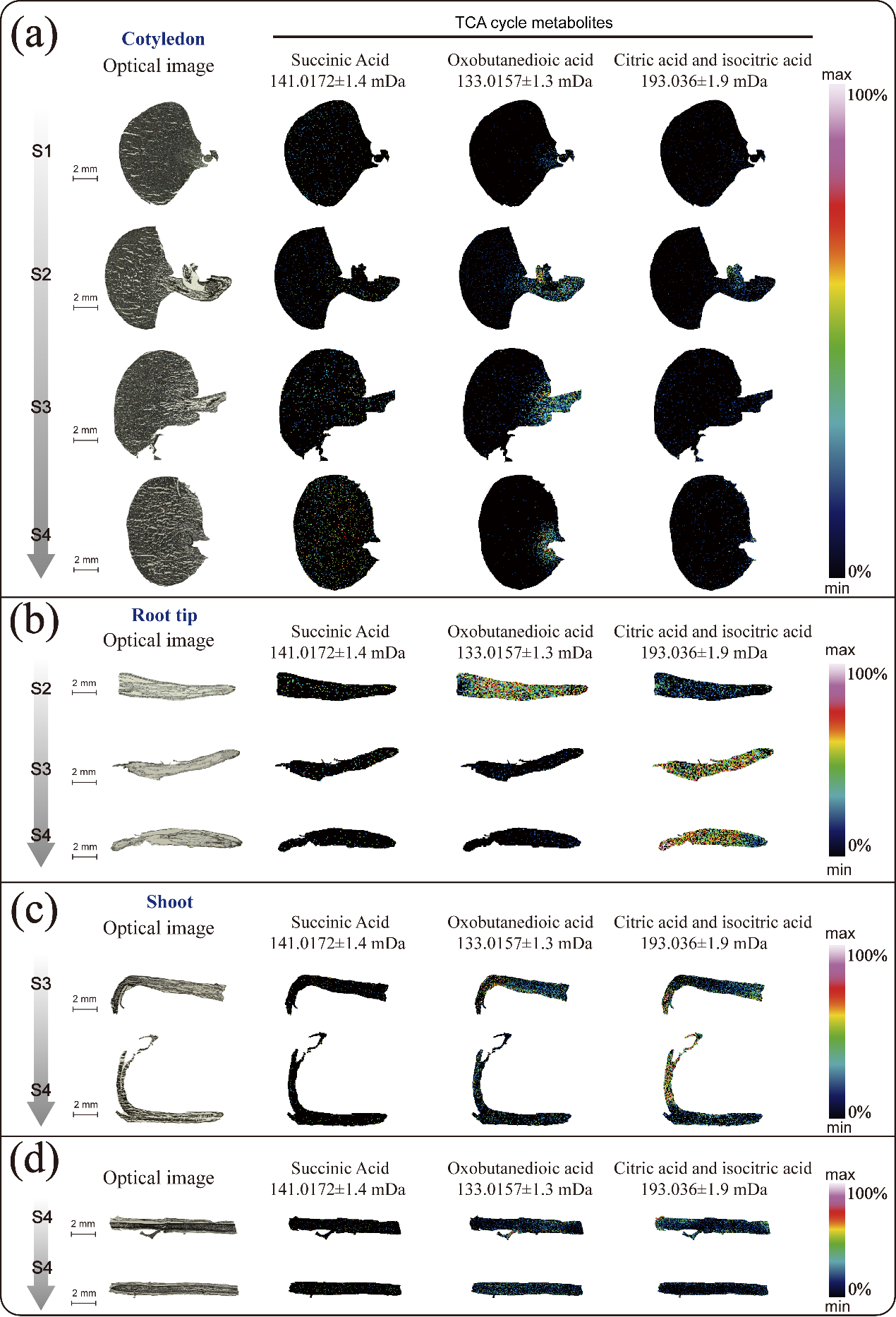


**Figure S8**. Spatial mass spectral distribution results of some metabolites in TCA. Optical images (left) and ion distribution images (right) of succinic acid, oxobutanedioic acid, and citric/isocitric acids in the cotyledons(a), root tip(b), epicotyl(c), and the middle of epicotyl and hypocotyl (up) and root maturation zones (down) (d) at different stages. The color scale goes from blue to red, representing a sequential increase in the content of the target substance in the region.
